# Supplementary material for: Documenting contributions to scholarly articles using CRediT and tenzing
Source: PLoS One. 2020 Dec 31;15(12):e0244611. doi: 10.1371/journal.pone.0244611 (PMC7775117; doi:10.1371/journal.pone.0244611)
Supplement: Attachment — Submitted filename: responseToReviewers.docx [file pone.0244611.s002.docx]

Dear Dr. Nwaru,

We greatly appreciate the five reviews for our manuscript. This provides a lot of value, and not only due to the constructive suggestions - thanks to PLoS’s option for open peer reviews, which we plan to take up, it also provides a public indication of the extent to which our manuscript was vetted and evaluated.

Response to comment by Napsi Szincsak of PLoS ONE

After the action letter, I received an email from Napsi Szincsak asking us to address the fact that PLoS ONE has specific guidelines on software sharing. We had already cited the Github page, where the source code is archived. We have now also added an MIT license (https://github.com/marton-balazs-kovacs/tenzing/blob/master/LICENSE.md).

Response to “journal requirements” comments

*-1. Please ensure that your manuscript meets PLOS ONE's style requirements, including those for file naming.*

We have adjusted the title page and believe the rest of the manuscript and files comport with the specified style.

-2. *In your Data Availability statement, you have not specified where the minimal data set underlying the results described in your manuscript can be found.*

That is correct – this is because there is no underlying data set, as there are no results described in the manuscript.

Response to reviewer 1’s comments

We appreciate that the reviewer believes the manuscript is interesting and that it achieves its aims of reviewing the state of author/contribution issues and presenting the R package and app for improving things.

Response to reviewer 2’s comments

We appreciate the reviewer’s praise for the manuscript as well as the multiple detailed “minor suggestions”.

*It would be interesting to see a bit more discussion raised concerning large(r)-scale projects and group authorship vis-à-vis the CRediT guidelines (e.g., Fontanarosa, Bauchner, & Flanagin, 2017;* [*https://jamanetwork.com/journals/jama/fullarticle/2667044*](https://protect-au.mimecast.com/s/dTaOCjZ1N7i03AAohMGnk_?domain=jamanetwork.com)*). Given that assigning the correct role to authors and collaborators has proven to be difficult, is crediting the entire research group as a whole (and thus no individual authors) perhaps a viable alternative? The specific roles (following, e.g., CRediT guidelines) of each author could then be specified on the group’s website, for instance.*

We agree that group (or consortium, as it is sometimes called) authors is a viable alternative to contributor schemes such as CRediT and use of them has become a strong tradition in some fields, such as genomics. Documenting CRediT information on the group’s website, however, would not be considered a great solution as websites are typically not as permanent as journal article records. We were not aware of the JAMA editorial the reviewer provided, which is helpful, so we have added a paragraph on this with that citation to this section.

*It is unfortunate that the “lead”, “equal”, and “supporting” specifications are not more widely adopted, and thus not a feature of tenzing. While I understand your reasons, I hope that you may add such a feature in coming releases. Perhaps showing how it can be done in practice will lead to faster adoption?*

Indeed, we do think that this is very important to specify in some projects, see some of our discussion here <https://github.com/marton-balazs-kovacs/tenzing/issues/15> . As mentioned at the discussion, there is presently no JATS (the underlying machine-readable metadata) specification in CRediT for lead vs. equal vs. supporting, but we expect that the group at the National Information Standards Organization (NISO) presently working on CRediT will address this, which will lead us to move this feature up in the development plan.

*I tested the Shiny app… The papaja YAML output fails to retrieve all roles; all roles are stated as "Conceptualization".*

This error has since been fixed.

*I found the code to be clean, consistent, and nicely commented*

We appreciate your gracious comment.

*I have two feature suggestions that may encourage quicker adoption by users:

(1) Allow users to create a template from scratch within the Shiny app (thus not having to download a template, edit it, and then upload it). It's a minor issue, but it is always nice to avoid having to move from one program to another. This could be done on a separate tab or page, and the final sheet could shown as a datatable, or similar, to allow editing within the browser (similar to the “Show infosheet” feature, but with editing capabilities). A download button would allow users to save the sheet locally, if need be.*

We have been discussing this feature, but implementing spreadsheet functionality within Shiny is currently beyond our programming resources as well as, possibly, the Shiny hosting infrastructure we have access to. However, we have been exploring the feasibility and usability of users entering into Tenzing the Google Doc URL for their contributorship spreadsheet so that users do not need to download and upload a template.

*(2) It would be helpful to allow for more than two affiliations. Perhaps this could be a dynamic variable?*

Yes, we have been working on this feature for a while, actually, trying different formats for the interface to avoid creating major usability problems. We hope to have it implemented in the next few months.

*• Line 49, missing abbreviation (ISWC) following mention of International Standard Musical Work Code
•Line 70, CRediT is misspelled as CrediT.
• Line 169, which is misspelled as whichh.*

Thanks for pointing these out – we have fixed them.

Response to Reviewer 3’s comments

*The authors describe a very important tool that appears to solve an emerging problem about authorship and contributions to scholarly articles. Their recommended tool is useful, and would help to initiate change in the way in which scientist conduct and report research/outputs.*

Thank you for the positive comments about *tenzing*.

*one challenge that has not been well elucidated is how to fairly document the depth of involvement by an author per category.*

We agree that this remains a big challenge. The CRediT standard itself has a way to partially address it, by allowing indication of whether a contributor made a “leading”, “equal”, or “supporting” role, but it is not yet fully implemented by CRediT: There is presently no JATS (the underlying machine-readable metadata) specification in CRediT for lead vs. equal vs. supporting, but we expect that the group at the National Information Standards Organization (NISO) presently working on CRediT will address this, which will likely prompt us to move this feature up in the development plan (see some of our discussion here <https://github.com/marton-balazs-kovacs/tenzing/issues/15>).

Response to Reviewer 4’s comments

*The topic itself is interesting and scientifically relevant, and could be suitable for the scope of the journal. Some minor comments for the authors:*

Thank you for the positive comments.

*- The manuscript still needs editing and language revision.*

We have made many edits to improve the wording, as can be seen in the track changes version.

*- The manuscript lacks some important references, for example, in the “introduction” section and “how journals are using CRediT”.*

Thank you for pointing this out - we have now added several references to these sections.

*- The facility of PLoS journals can be provided as a separate figure e.g. Figure 2 (as for eLife)*

Done.

*- Instead of links to external blogs, I recommend authors to provide tables describing different features of CRediT e.g. limitations and applicability.*

Thank you for the suggestion. We have added a table describing the basic features of CRediT (Table 1), which also provides some indication of CRediT’s limitations.

Response to Reviewer 5’s comments

*I would appreciate having more information on how tenzing is advantageous? In what way is this method more superior to existing ones?*

One reason that this aspect of the manuscript may have appeared lacking is because we don’t know of any alternative methods to *tenzing*, in the sense of an easy to use (no programming) tool to facilitate CRediT reporting. To address the reviewer’s comment, however, we have added more references to somewhat related approaches, and have added a paragraph about “authorship grids” to better explicate the unique utility of *tenzing*.

*Is there any possibility to trace the changes researches make in tenzing?*

The researchers enter the contributorship information into a Google Sheet (or they can download that and make changes in a spreadsheet of their choice). Google provides a version history that can be reviewed, for example to understand how contributorship roles evolve during the course of the project.

*Many different components of the same problem emerge in most parts of the manuscript, which illustrates how multidimensional the question is. At the same time, it would be clearer if authors were to focus on a limited inquiry, what part of the problem do authors want to solve? Another possible alternative is to choose a different article type.

I find the structure of this article unclear. The authors should clarify the following sections: problem definition, proposed solution, discussion, conclusion.

The main strength of this manuscript is that it addresses a timely question with great potential for improvement. I agree with the authors that there is a need to develop methods and strategies to ensure transparency in the research were contribution plays a significant part. But still, there is an unresolved question regarding what approach would solve the particularly chosen part of the issue.*

We tentatively agree that doing nearly a complete rewrite of the manuscript with the structure proposed by the reviewer should make it clearer to some readers. However, given that the other four reviewers did not suggest that a rewrite was necessary, we prefer not to make too many changes that might perturb the manuscript in a way that might make the other reviewers less happy. We also agree that ideally, an argument could be made that *tenzing* is the optimal way to address the needs of researchers and the interests of science broadly. However, we make no such claim in the manuscript – instead we aimed only to explain some of the issues and that *tenzing* partially addresses some of them. We think that the limited rewrite that we have done makes that a bit more clear.
