# Supplementary material for: Documenting contributions to scholarly articles using CRediT and tenzing
Source: PLoS One. 2020 Dec 31;15(12):e0244611. doi: 10.1371/journal.pone.0244611 (PMC7775117; doi:10.1371/journal.pone.0244611)
Supplement: Attachment — Submitted filename: Reviewer comments.docx [file pone.0244611.s001.docx]

Title: Documenting contributions to scholarly articles using CRediT and tensing

Revised by: Madar Talibov

The article summarizes advantages and limitations of traditional methods (e.g. “Author Note”, “Author information”) which have been used by scientific journals for many years. Then the authors briefly describe Contributor Role Taxonomy (CRediT) which was developed in 2014. CRediT tool has an advantage over existing methods by being more detailed and structured. CRediT defines 14 different types of author contributions. If this method would be widely adopted by publishers, it could help to provide a better documentation of the contribution of researchers. Finally, the paper presents the new web app and R package (*tenzing*) which helps to facilitate CRediT tool.

The authors aimed:

1. To revise the current state of how journals use CRediT tool for documentation of author contribution and difficulties associated with the use of CRediT during submission process.

2. To present R package and web app (*tenzing*) which they developed to overcome these difficulties.

In my opinion, the authors have achieved both aims. The paper summarizes how some journals are currently using new author contribution documentation tool CRediT, and explains difficulties of using it based on illustrative examples. Free web app and R package *tenzing* developed by authors achieves its purpose. Figures included in the paper are very informative and help to understand how *tenzing* package works.

I find the paper very interesting and I think it would be beneficial to replace traditional author contribution methods by more sophisticated methods such as CRediT in the future.

If the structure of the paper meets the requirements of PLUS ONE journal, then I would suggest accepting the paper.
